# Impact of Anthropogenic Factors on the Diversity of Microbial Communities of PM10 Air and PM100 of *Tilia* L. Phylloplane in an Urban Ecosystem

**DOI:** 10.3390/biology13120969

**Published:** 2024-11-24

**Authors:** Olesya I. Sazonova, Anastasia A. Ivanova, Anna A. Vetrova, Anton N. Zvonarev, Rostislav A. Streletskii, Viacheslav I. Vasenev, Vladimir A. Myazin, Ksenia I. Makhinya, Ekaterina V. Kozlova, Maria V. Korneykova

**Affiliations:** 1 Federal Research Center “Pushchino Scientific Center for Biological Research of the Russian Academy of Sciences”, 142290 Pushchino, Russia; 2 Laboratory of Ecological Soil Science, Faculty of Soil Science, Lomonosov Moscow State University, 119991 Moscow, Russia; 3 Soil Geography and Landscape Group, Wageningen University, 6707 Wageningen, The Netherlands; 4 Agrarian and Technological Institute, People’s Friendship University of Russia (RUDN University), 117198 Moscow, Russia; 5 Institute of North Industrial Ecology Problems Subdivision of the Federal Research Center “Kola Science Centre of Russian Academy of Science”, 184209 Apatity, Russia

**Keywords:** particulate matter, metabarcoding, bacterial communities, fungal communities, metals, PAHs

## Abstract

Identifying the relationship between the microbiomes of urban dust particles from different biotopes is important because the state of microorganisms can be used to assess the quality of the environment. The aim of this work was to determine the distribution and interaction patterns of microorganisms of dust particles in the air and on leaf surfaces. Metabarcoding of bacterial and fungal communities, PAH, and metal content analyses and electron microscopy were used in this work. The results obtained allowed us to characterise the biological and chemical components of the dust particles. Some bacterial and fungal genera were correlated with benzanthracene, fluoranthene, and Cu, Ni, Co, Zn, and Mn contents. Bacterial communities were found to be more sensitive to all the pollutants studied. PM10 microbial communities circulated between biotopes and study areas due to air flows, as evidenced by the presence of similar ASVs in fungi and bacteria. The results could help to understand the effects of climate change and anthropogenic activities.

## Simple Summary

The air microbiome is one of the most poorly characterized microbial communities of environmental objects. It has been suggested that leaves are indicators of air quality. In the present work, the taxonomic and functional diversity of bacterial and fungal communities, PAHs, and metal contents in PM100 of leaves and PM10 of air collected from two zones with different levels of anthropogenic pollution were compared. Dust particles found on the surface of leaves and air filters were also compared using scanning electron microscopy. Our data showed that air pollution had a greater effect on phylloplane bacterial communities. The effect of air pollution on bacterial communities could facilitate their use as indicators for monitoring atmospheric conditions. At the same time, bacterial organisms are performers whose role in biotechnological methods of air purification is inevitably important.

## 1. Introduction

Particulate matter (PM) is a general term for a type of air pollutant that is a complex mixture of particles in the air. PM is generated by various natural events and anthropogenic activities [1]. PM10 and PM2.5 (particles with diameters up to 10 µm and 2.5 µm, respectively) are part of the urban air pollution components. These particles are characterized by their long persistence in the atmosphere as part of aerosols and their ability to travel over long distances [2,3]. Particulate matter varies in its chemical composition, reactive properties, and decay time [1]. The degree to which PM impacts human health is directly related to its size, surface area, quantity, and composition [4]. Changes in human health under the influence of air pollutants vary widely, including symptoms such as nausea, respiratory distress, skin irritation, swelling, and cancer [5,6,7]. This range of adverse effects is determined not only by the composition of the pollutants in the atmosphere and their dose but also by the duration of exposure. The level of anthropogenic pollution has an impact on the composition of urban dust and its fractions. The highest concentrations of PM10 particles are found in industrial areas, where dust concentrations are increased by industrial and construction sites, road abrasion, and vehicle emissions [2].

The following strategies have been identified as the most effective for managing air quality: controlling different pollutants, switching to renewable energy sources, increasing the use of electricity as an alternative to traditional sources of PM emissions (such as conventional gas or fuel oil combustion), and implementing green infrastructure in urban areas [8]. The latter is due to the fact that various plants are effective at trapping atmospheric particles. Plants can remove pollutants by filtration (deposition) of large and fine PM on their leaves, branches, and stems, or by uptake of gases through leaf stomata. Filtration and uptake rates are influenced by leaf characteristics (size, shape) and factors such as location and meteorological parameters such as precipitation [9]. Micromorphological features of leaves (lint, stomata, and grooves on the leaf surface) can increase their roughness, resulting in more effective dust retention. Weerakkody et al. [10] showed that PM concentrations were higher on adaxial than on abaxial leaf surfaces in all plant species studied. Precipitation can wash PM deposited on leaves into drainage channels and/or soil [11]. In turn, PM can adversely affect plant growth by interfering with processes such as transpiration and photosynthesis, blocking stomata, and causing chronic leaf damage [12].

Various polycyclic aromatic hydrocarbons (PAHs) are considered to be the most toxic components of PM. Since PAHs are hydrophobic, their deposition on the leaf surface is mainly dry (in gaseous or PM-bound form). The stereomicrostructure and hydrophobicity of the leaf surface can influence the uptake of organic pollutants. However, there are few studies on this aspect. The uptake capacity of PAHs differs between plant species [13]. On the leaf surface, stomata and cuticular plates are the main channels through which gaseous PAHs preferentially penetrate, whereas PAHs adsorbed on particles are mainly deposited on the leaf surface, where they are subsequently desorbed from the particles [14]. Once accumulated in the leaves, PAHs are eventually transferred from the leaves to the soil through the leaf fall process. So, leaf microbial communities, PAHs, and other pollutants accumulated on the surface and in leaf tissues enter the soil mainly in the autumn. PAHs can be degraded by microorganisms, which are considered to be one of the most important tools for the removal of PAHs from the environment.

As heavy metals are known to be non-biodegradable, these pollutants are a constant threat to the environment. Prolonged exposure to heavy metals reduces plant functionality by disrupting chlorophyll synthesis, affecting respiration and photosynthesis, and inducing the formation of reactive oxygen species [15]. At present, there are few publications investigating the response of phylloplane microorganisms to heavy metal stress. In Khan et al. [16], *Bacillus amyloliquefaciens* isolated from leaves demonstrated its ability to absorb heavy metals and consequently reduce the concentration of metal ions in the microenvironment. Microorganisms, as an essential part of ecosystems, have been shown to help plants cope with heavy metal stress through complexation, transformation, and detoxification mechanisms [17]. For example, proteobacteria can reduce heavy metal toxicity through biotransformation or utilization of various organic substances for survival and improve carbon and nitrogen cycling, directly or indirectly promoting plant growth and the nutritional status of plants [12].

Thus, the phyllosphere microbiome plays a critical role in improving leaf function, resistance to pathogens and environmental stresses, and increasing plant productivity [12,18]. Sources of phyllosphere microbiota are soil, seeds, and airborne PM [18]. Soil microorganisms can invade roots and move through vascular tissues to the leaves. The phyllosphere microbiota is quite diverse and variable. Many environmental factors (sunlight, UV radiation, and precipitation), insect/pathogen contamination, fertilizer application, and soil type actively influence the formation of the microbiota [19]. Microstructural characteristics of the leaves can also influence the microbial communities of the phylloplane [20,21].

Despite the great taxonomic diversity in nature, the bacterial phyllosphere communities of seed plants studied so far have been dominated by a few phyla such as *Pseudomonadota*, *Actinomycetota*, *Bacteroidota*, and *Bacillota*. In several eudicot species studied, the phyllosphere microbiota was dominated by bacteria of only a few genera; for example *Sphingomonas* (class *Alphaproteobacteria*/phylum *Pseudomonadota*), *Methylobacterium* (class *Alphaproteobacteria*/phylum *Pseudomonadota*), and *Pseudomonas* (class *Gammaproteobacteria*/phylum *Pseudomonadota*). Similar bacterial genera have been found in the phyllosphere of monocotyledonous plants such as rice, miscanthus, and plantain. Numerous bacterial families of alpha-proteobacteria, including *Sphingomonadaceae*, *Beijerinckiaceae*, and *Acetobacteraceae*, were abundant in the phyllosphere microbiome of woody species representing gymnosperms and angiosperms plants in temperate and subtropical forests. In particular, the widespread distribution of *Sphingomonas* and *Methylobacterium* as members of the phylloplane microbiota has been linked to their adaptation to the low amount of nutrients available at the leaf surface [22].

Li and co-authors [23] showed that phylloplane bacterial communities from urban roads with high dust exposure and leaves collected from other parts of the city were significantly different. The relative abundance of microorganisms was highest on leaves from the park area, which was explained by the positive effect of humidity on growth. The dominant bacteria were *Gammaproteobacteria*. In Dang et al. [12], it was shown that dust on the leaf surface had a significant impact on the community of foliar microorganisms. At the same time, the diversity of the phyllosphere microbial community was significantly lower in the industrial zone than in the control zone. In Qian et al. [24], the α-diversity of phylloplane bacterial communities was studied to assess their response to the presence of heavy metals. The results of the study showed that heavy metals suppressed the most dominant bacteria, providing more habitat for other sensitive bacteria and improving the richness and diversity of the phylloplane bacterial community. In turn, phylloplane can be a source of airborne circulating microorganisms. For example, Awad [25] showed that plants are the source of *Alternaria* in the air; *Aspergillus*, *Penicillium*, and *Cladosporium* have been linked to environmental urbanization. Despite the scientific work that has appeared in recent years, the question of how the microbiome of the phylloplane changes under the influence of PM deposited on the leaf surface from the atmosphere remains insufficiently studied.

The aim of this study was to investigate the differences in the structure and function of the microbial communities on the surface of *Tilia* L. leaves between the campus zone and the urban construction zone under the impact of atmospheric PM particles, including PAHs and heavy metals.

## 2. Materials and Methods

### 2.1. Sampling Company

#### 2.1.1. Sites Description and Sampling Period

Two biotopes were investigated in this study: air and phylloplane. Air PM10 and leaf surface PM100 samples were collected in the RUDN area (Moscow) in September 2023: inside the campus area (campus) and outside the campus area in the vicinity of the zone of high-rise buildings under construction and the road (construction zone). These zones were selected to assess the effects of anthropogenic pollution levels on PM10 and PM100 microbiomes. The geographical coordinates of the sampling sites were as follows: 55.64909° N, 037.50158° E (woody plant growing on the campus) (1, Figure S1), 55.64934° N, 037.50189° E (air PM10 sampled in the campus zone using an air sampler) (2, Figure S1), 55.64850° N, 037. 50677° E (woody plant growing near the construction zone) (3, Figure S1), and filter road 55.64866° N, 037.50663° E (air PM10 sampled in the construction zone) (4, Figure S1).

#### 2.1.2. Sample Collection

To sample PM10 from atmospheric air, SKC IMPACT samplers (Dorset, UK) were used with a flow rate of 10 L/min by filtering the air through polycarbonate filters (47 mm diameter, 0.8 μm pore size). The samplers were placed at a height of 3 m above ground level. This correlated with the height at which the leaves were sampled. Similarly, PM10 samples were collected for PAH content analysis, air filter surface microscopy, and microbiome analysis by metabarcoding. Sampling was conducted over a period of 8 days. Filters were changed every 2 days to eliminate the possibility of pore-clogging, as recommended by Ferguson et al. [26]. Thus, four polycarbonate filters were obtained from each sampler and pooled together for further analysis.

The High Spatial Resolution Sampler (HSRS, Fai Instruments, Rome, Italy) was used to estimate the metal content of PM10 in the air. In this case, the flow rate was at 2 L/min. Particles were sampled on polytetrafluoroethylene (PTFE) membrane filters (47 mm diameter, 0.45 μm pore size). Samples of PM10 were taken from each zone over a period of 8 days. In contrast to the polycarbonate filter used for metabarcoding and PAH analysis, the PTFE membrane filters remained unchanged throughout the PM10 collection period.

Samples of PM10 on filters selected for all analyses were placed in hermetically sealed containers and transported at 4 °C. Upon delivery, the filters were stored at −20 °C until immediate analysis.

For PM sampling from leaf surfaces (PM100), leaves were collected from *Tilia* L. growing in close proximity to the atmospheric PM10 sampling sites. Leaves were collected randomly from the entire tree canopy at a height of 2.5–3 m above ground level in sealed plastic containers and transported to the laboratory at 4 °C. PM100 samples were immediately prepared for further analyses. In parallel, area of leaves was counted using ImageJ 1.54g software (developed at the National Institutes of Health and the Laboratory for Optical and Computational Instrumentation (LOCI, University of Wisconsin, Madison, WI, USA)).

To analyze the microbiome associated with PM100, leaves with a total area of approximately 1000 cm^2^ were placed in 300 mL of physiological solution and stirred vigorously on an orbital shaker at 200 rpm for 15 min. The resulting solution was filtered through nylon bellows with a pore size of 100 µm to remove large inclusions. The filtrate was then passed through vacuum funnels containing polyethersulfonate filters with a pore diameter of 0.22 μm. Thus, one filter with PM100 was obtained for each zone. The filters were removed and frozen until DNA extraction.

To analyze PAHs in PM100, dust particles were washed off the leaves with deionized water. For this purpose, leaves with a total area of about 300 cm^2^ were placed in a flask with 30 mL of deionized water and stirred vigorously at 200 rpm for 15 min. The resulting suspension was transferred to a 100 mL glass flask, and 30 mL of methylene chloride (France) were added to obtain extracts for further analysis of the PAHs by high-performance liquid chromatography (HPLC).

To analyze the metal content in PM100, dust particles were washed off the leaf surface with deionized water. For this purpose, leaves with a total area of approximately 300 cm^2^ were placed in a flask containing 50 mL of deionized water and stirred vigorously at 200 rpm for 15 min. The resulting suspension was collected in glass vials and incubated at 65 °C for 72 h until the water evaporated. Evaporated deionized water was used as a control. The samples were used for further analysis by inductively coupled plasma optical emission spectrometry (ICP-OES).

### 2.2. DNA Extraction and 16S/ITS Metabarcoding

Four polycarbonate filters containing atmospheric PM10 from each zone were pooled together for further DNA extraction using the DNeasy PowerWater Kit (Qiagen, Germany) according to the manufacturer’s protocol. This kit was also used to isolate microbial DNA from the surface of polyethersulfonate filters. The DNA was eluted with 50 µL of EB solution.

The quality and quantity of isolated DNA were analysed on a Qubit 2.0 spectrophotometer (Invitrogen/Life Technologies, Waltham, MA, USA). The DNA samples obtained were used as a matrix for polymerase chain reaction (PCR) with universal primer pairs. The following primers were used for amplification of hypervariable V3–V4 regions of the bacterial 16S rRNA gene: 341F 5′-CCTACGGGNGGCWGCAG-3′ (Forward) and 805R 5′-GACTACHVGGGTATCTAATCC-3′ (Reverse) [27]. ITS1 5′-TCCGTAGGTGAACCTGCGG-3′ (Forward) and ITS4 5′-TCCTCCTCCGCTTATTGATTGATGATGC-3′ (Reverse) primers targeting the ITS1-5.8S-ITS2 region of the rRNA gene cluster were used to analyze the fungal community [28]. Amplicon libraries for bacteria and fungi were prepared separately using the Nextera XT library preparation technology (Illumina, San Diego, CA, USA); paired-end sequencing (2 × 300 cycles) was performed on the Illumina MiSeq platform according to the manufacturer’s protocol (Illumina, San Diego, CA, USA). PCR, library preparation, and sequencing were performed by Sequentia Biotech SL (Barcelona, Spain).

### 2.3. Bioinformatic Analysis

The DADA2 package in R [29] was used to obtain amplicon sequence variants (ASVs) from paired-end demultiplexed reads of two amplicon libraries (16S rRNA and ITS1-5.8S-ITS2). After primer removal, forward and reverse reads were truncated as indicated by sequence quality-control results. For 16S rRNA, the forward reads were trimmed to 280 bp, and the reverse reads were trimmed to 185 bp; for the ITS1-5.8S-ITS2 region of fungi, the final read lengths were 250 bp and 180 bp, respectively. After merging the forward and reverse reads, chimeric sequences were identified and removed. For taxonomic classification of the merged reads, we used the SILVA databases v. 138.1 (updated 10 March 2021) for 16S rRNA amplicons and the UNITE ITS database v. 10.0 (updated 4 April 2024) for ITS1-5.8S-ITS2 amplicons [30,31]. Reads that could not be assigned to bacteria or fungi, and those that could not be classified at the kingdom level, were removed from further microbial community analyses.

### 2.4. Analyses of Chemical Components

#### 2.4.1. Analyses of PAHs

PAHs were analyzed by high-performance liquid chromatography (HPLC) using an Agilent 1260 system (Santa Clara, CA, USA) with a fluorescence detector. Sixteen PAHs were analyzed (anthracene, pyrene, benz[a]anthracene, chrysene, benz[e]pyrene, benz[a]pyrene, benz[a]pyrene, dibenz[ah]anthracene, naphthalene, acenaphthene, fluorene, phenanthrene, benz[k]fluoranthene, benz[ghi]perylene, fluoranthene, benz[b]fluoranthene, and in-deno[cd]pyrene) according to US EPA [32]. The detection limit of PAHs in these samples, obtained by a combination of PAH extraction and HPLC methods, was 0.05 µg/kg. Extraction rates ranged from 90 to 97%. PAH Calibration Mix (Merck, Darmstadt, Germany) was used as a standard. The components were quantified by the absolute calibration curve method.

#### 2.4.2. Analyses of Metals

The concentrations of Pb, Zn, Co, Cd, Cu, Ni, Cr, Fe, Mn, V, Sr, Ca, and Mg in the samples were measured using an ICP-OES Avio2000 (PerkinElmer, Waltham, MA, USA). The chemical elements were selected to represent different sources of contamination: elements of natural origin, elements of traffic origin, and elements of industrial origin [33].

### 2.5. Scanning Electron Microscopy

The surfaces of the leaves and filters were examined with scanning electron microscopy (SEM) JSM-6510LV (JEOL, Tokyo, Japan). Samples of the leaves and filters placed on aluminum table were fixed in glutaraldehyde vapor for 24 h at 4 °C and postfixed in OsO_4_ vapor for 3 h at 20 °C. After dehydration in propylene oxide vapor, the samples were coated with gold (Fine Coat Ion SputterJFC-1100, Tokyo, Japan) and examined under a scanning microscope JSM-6510LV (JEOL, Tokyo, Japan).

### 2.6. Statistical Analysis

All preliminary statistical analyses were performed using R v4.2.1 (R Development Core 2022, Vienna, Austria), a programming language for statistical processing, and the associated packages listed below. The ASV tables were rarefied to a minimal library using the *rarefy* from the *vegan* v2.6-2 R package to reduce the impact of sequencing depth on the assessment of differences between communities [34]. This parameter was 21,758 reads for the bacterial community and 42,450 reads for the fungal community. Five α-diversity indices (Chao1, Fisher, Simpson, Observed, and Shannon) were calculated using the *phyloseq* v1.40.0 R package [35]. The values obtained were used to assess the effect of anthropogenic pollution and zonality on their variation. Significant differences were calculated using the package *rstatix* v0.7.1 R; visualization was performed using the package *ggpubr* v0.5.0 R. Differences in the composition of both fungal and bacterial communities as a function of sample type (PM10 air or PM100 leaf surface) and urban zonality were analyzed by β-diversity analysis using the Bray–Curtis dissimilarity matrix at the species level. Dendrograms (separately for bacteria and separately for fungi) based on the Bray–Curtis dissimilarity matrix were also constructed to estimate β-diversity at the class level for the studied communities.

The online service Venny was used to construct Venn diagrams [36]. Heat maps and hierarchical clustering were performed in Morpheus [37].

To determine the relationship between the microbial community and metals, data were pre-ranked, and correlations were determined using the Spearman coefficient. Only statistically significant Spearman coefficients at *p* < 0.05 with Bonferroni correction were considered. The selected metals and genera of bacteria and fungi described above were used to perform redundancy analysis. The redundancy analysis (RDA) was performed in XLSTAT using R package 4.2.1.

In this work, we also evaluated differences between communities with respect to nutritional modes and putative microbial functions. For bacterial communities, we used the Functional Annotation of Prokaryotic Taxa (FAPROTAX) databases [38], and for fungal communities, we used the FUNGuild database [39]. Functional annotations of the communities were performed using Python v 3.12.4 (Python Software Foundation 2024, Wilmington, DE, USA). Post-processing visualizations were performed using the online resource Morpheus. To visualize the data obtained after processing the results of FUNGuild and FAPROTAX, the indicators were normalized according to the principle of min–max scaling. Pearson’s linear correlation coefficient was used to identify the relationship.

## 3. Results

The study involved the collection of airborne PM10 and dust PM100 from *Tilia* L. leaf surfaces to investigate differences in the structure and function of the bacterial and fungal communities associated with these PMs under anthropogenic stress. To achieve this aim, the following tasks were performed: microscopy of the surface of polycarbonate air filters and the adaxial side of leaves, qualitative and quantitative assessment of the content of PAHs and metals in PM10 and PM100, taxonomic characterization of the bacterial and fungal communities, and statistical analysis of possible interactions between pollutants and representatives of the microbial community.

### 3.1. Scanning Electron Microscopy of the Adaxial Side of Leaves and the Surface of Air Filters

**Figure 1:**
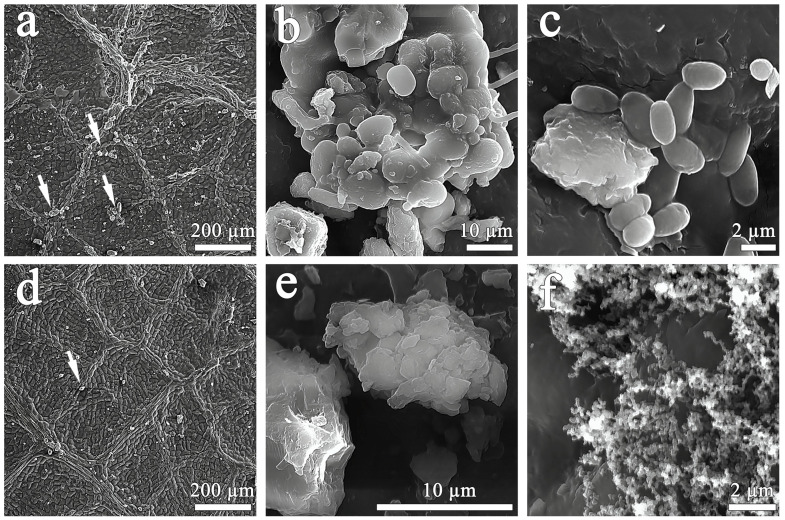
Scanning electron microscopy of the adaxial surface of leaves in the campus zone (**a**–**c**) and in the construction zone (**d**–**f**). In (**a**,**d**), the arrows indicate the detected PM.

Scanning electron microscopy was conducted on the adaxial side of the leaves collected in the area of the RUDN campus and near the road bordering the campus (Figure 1). As shown in Figure 1, the leaves of *Tilia* L. had an irregular relief surface formed by microveins. Particulate matter of various sizes accumulated mainly near and directly on the veins. Apparently, they served as a barrier for PM and microorganisms. Trichomes and stomata were not detected on the adaxial side of *Tilia* L. leaves. PMs were mostly irregular in shape and had a rough surface. Leaves collected in the campus area (Figure 1a) had higher levels of particulate matter than leaves collected near the road (Figure 1d). The magnification of particles detected on leaves in the campus area and near the construction zone are shown in Figure 1b,c and Figure 1e,f, respectively. In Figure 1b,c, oval cells were clearly visible. Therefore, we assumed a predominance of the biological component associated with PM100 on the campus leaves (Figure 1b,c) in contrast to the leaves sampled near the construction zone (Figure 1e,f). We also observed the presence of soot particles (Figure 1f) in the form of chain-like clusters on the leaves of the construction zone.

**Figure 2:**
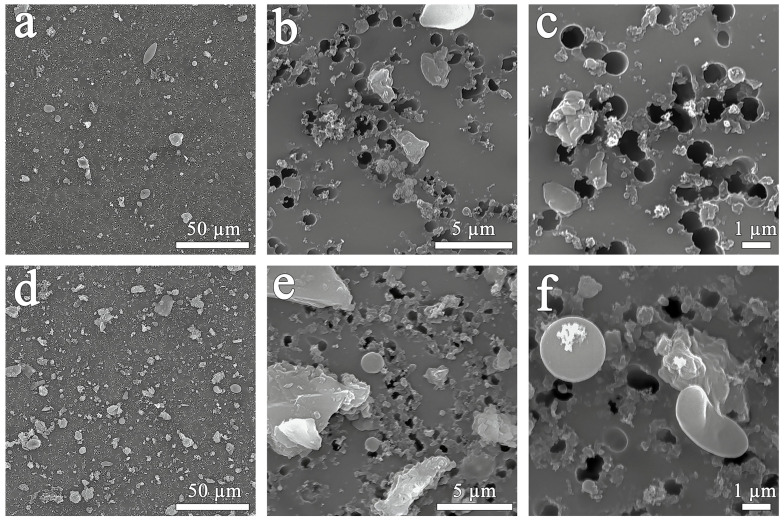
Scanning electron microscopy of the filter surface after PM10 sampling in the campus zone (**a**–**c**) and the construction zone (**d**–**f**).

Air filter surfaces were photographed using a scanning electron microscope after 8 days of air sampling in the RUDN campus zone (Figure 2a,c) and in the construction zone adjacent to the campus (Figure 2d,f). As shown in Figure 2a,d, the air in the construction zone contained more particulate matter than the air in the campus zone. We visually counted the amount of PM10 on both filters per 50 μm^2^. The counts of PM10 in the campus zone (Figure 2a) were four times lower than those in the construction zone (Figure 2d). The filters showed smooth spherical particles of various diameters.

### 3.2. Diversity of Bacterial and Fungal Communities

After sequencing and subsequent processing of the reads, we identified a total of 2528 amplicon sequence variants (ASVs) for the bacterial communities and 783 for the fungal communities of PM10 and PM100.

#### 3.2.1. α-Diversity Indices

**Figure 3:**
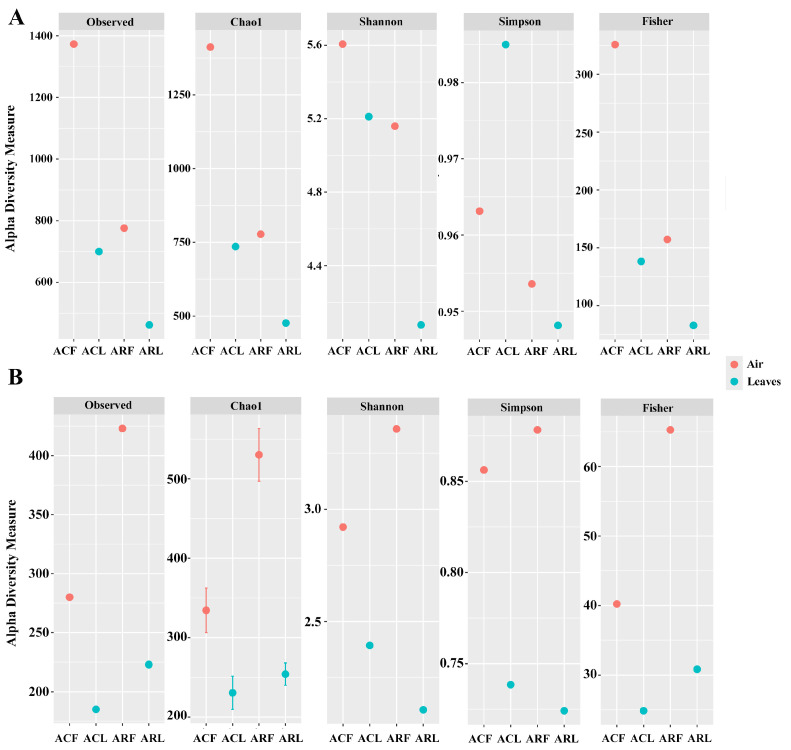
Alpha diversity indices of bacterial (**A**) and fungal (**B**) communities PM10 of air and PM100 of leaf surfaces. ACF—PM10 of air from campus zone, ACL—PM100 of phylloplane from campus zone, ARF—PM10 of air from construction zone, ARL—PM100 of phylloplane from construction zone.

Alpha diversity indices of bacterial (A) and fungal (B) communities were calculated based on the obtained ASVs (Figure 3). It can be noted that almost all indices were characterized by higher alpha diversity values of bacterial and fungal communities in the case of air PM10. The exception was the Simpson index value of the bacterial community PM100 of leaves sampled in the campus zone. In addition, the alpha diversity indices of bacterial communities associated with PM collected from the same biotope but from different zones were higher in the case of samples collected on the campus. The opposite trend was observed for fungal communities, except for the Shannon and Simpson indices of PM100 communities from leaf surfaces. Low values of Shannon and Simpson indices in the bacterial and fungal communities of PM100 from the construction zone indicated that these communities were not very diverse and had the least complex structure compared to the other samples.

The Fisher index in biocenoses is characterized as follows: a high α value means a large number of rare species (with low density) compared to common species with high population density. Conversely, a low α value means that the number of common species in the biocenosis is greater than the number of rare species, i.e., the biocenosis is characterized by a low degree of species diversity. A similar phenomenon was found for the bacterial and fungal communities of phylloplane PM100 (Figure 3). These communities were also characterized by low species richness.

#### 3.2.2. Taxonomic Characterization of Bacterial and Fungal Communities

##### Taxonomic Characterization of Bacterial Communities

**Figure 4:**
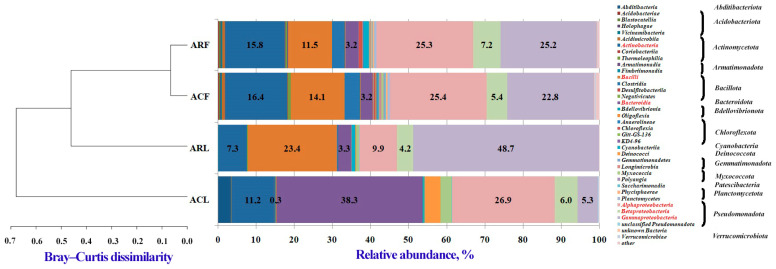
Distribution of the most abundant bacterial classes identified in PM10 and PM100 collected in two functional zones of Moscow. Unclassified classes and classes with relative abundance ≤ 0.1% were considered “unclassified” and “other”, respectively. Clustering analysis of the class-level communities based on the calculation of the pairwise Bray–Curtis dissimilarity matrix was also reported. ACF—PM10 of air from campus zone, ACL—PM100 of phylloplane from campus zone, ARF—PM10 of air from construction zone, ARL—PM100 of phylloplane from construction zone. Dominant classes in the sample bacterial communities are shown in red.

Twenty-four bacterial phyla were identified. The phylum *Pseudomonadota* (38.3–62.91%) had the highest relative abundance in the bacterial communities of the investigated samples, followed by *Bacteroidota* (3.23–38.29%), *Bacillota* (0.3–23.72%), and *Actinomycetota* (7.63–18.06%). In total, 72 bacterial classes were identified (Figure 4, Table S1).

The dominant classes in the bacterial communities of the samples were *Actinobacteria* (phylum *Actinomycetota*) (7.35–16.43%), *Bacilli* (phylum *Bacillota*) (0.26–23.42%), *Bacteroidia* (phylum *Bacteroidota*) (3.17–38.29%), *Alphaproteobacteria* (phylum *Pseudomonadota*) (9.92–26.87%), *Gammaproteobacteria* (phylum *Pseudomonadota*) (5.28–48.74%), and *Betaproteobacteria* (phylum *Pseudomonadota*) (4.21–7.23%).

Furthermore, the class *Bacteroidia* in the PM100 samples of *Tilia* L. leaves of the campus zone (sample ACL in Figure 4) had the highest percentage of abundance (38.3%) compared to other classes in the community, as well as compared to the abundance of this class in the communities of the other samples (3.2–3.3%). Simultaneously with the increase of *Bacteroidia*, a low relative abundance of the class *Bacilli* (0.3%) was observed in the PM100 samples of *Tilia* L. leaves on the campus. The opposite trend (increase in the abundance of the class *Bacilli* to 11.5–23.4%) was observed in the other samples. Also, in the PM100 of leaves of the campus zone, we found the presence of significant numbers of representatives of classes such as *Deinococci* (phylum *Deinococcota*) (4.1%), *Myxococcia* (phylum *Myxococcota*) (2.9%), and *Abditibacteria* (phylum *Abditibacteriota*) (3.4%) compared to these classes in other samples. The bacterial community of sample PM100 from the leaves of the construction zone was characterized by the highest relative abundance of the classes *Gammaproteobacteria* (48.7%) and *Bacilli* (23.4%), with a simultaneous decrease of other classes.

The bacterial communities of the PM10 samples from the campus zone and the construction zone were characterized by similarity in qualitative composition and relative abundance of the identified classes. The dendrogram based on the Bray–Curtis dissimilarity matrix also showed the similarity of these communities at the class level. In addition, the bacterial community of PM100 from leaves of the campus zone was significantly different from the other three communities studied (Figure 4).

A total of 157 bacterial orders were identified (Figure S2, Table S1). The orders with the highest abundance in the communities of the studied samples were *Bacillales* (phylum *Bacillota*, class *Bacilli*) (0.1–9.9%), *Burkholderiales* (phylum *Pseudomonadota*, class *Betaproteobacteria*) (4.1–7.2%), *Cytophagales* (phylum *Bacteroidota*, class *Bacteroidia*) (1.4–37.4%), *Enterobacterales* (phylum *Pseudomonadota*, class *Gammaproteobacteria*) (0.8–25.5%), *Lactobacillales* (phylum *Bacillota*, class *Bacilli*) (0.2–22.6%), *Micrococcales* (phylum *Actinomycetota*, class *Actinobacteria*) (3.7–9.3%), *Pseudomonadales* (phylum *Pseudomonadota*, class *Gammaproteobacteria*) (0.6–20.9%), *Rhizobiales* (phylum *Pseudomonadota*, class *Alphaproteobacteria*) (1.5–7.2%), *Rhodobacterales* (phylum *Pseudomonadota*, class *Alphaproteobacteria*) (0.7–7.4%), and *Sphingomonadales* (phylum *Pseudomonadota*, class *Alphaproteobacteria*) (4.0–15.8%).

At the order level, the bacterial communities of the PM10 air samples from the campus and the construction zone were similar to those at the class level, which was also reflected in the dendrogram based on the Bray–Curtis dissimilarity matrix (Figure S2). The orders *Pseudomonadales*, *Micrococcales*, and *Bacillales* had the highest relative abundance in these samples. Regarding the composition of the bacterial communities from the construction zone and the campus leaf dust samples, there was a clear difference at the order level (as well as at the class level). In the PM100 sample of leaves from the campus zone, the highest relative abundance of the order *Cytophagales* was observed (37.4%), while in all other samples, this value was 10 times lower. Another peculiarity of the bacterial community of PM100 of *Tilia* L. leaves from the campus was the high percentage of the orders *Abditibacteriales* (phylum *Abditibacteriota*, class *Abditibacteria*) (3.4%) and *Deinococcales* (phylum *Deinococcota*, class *Deinococci*) compared to the other three samples (PM100 of leaves from the construction zone and PM10 of air from the campus and the construction zone). The composition of the bacterial community of leaf samples collected near the construction zone differed from the other samples by an increased abundance of the orders *Enterobacterales* (25.5%), *Lactobacillales* (22.6%), and *Piscirickettsiales* (phylum *Pseudomonadota*, class *Gammaproteobacteria*) (3.9%). It should be noted that the orders *Pseudomonadales* and *Bacillales* had a very low relative abundance in the communities of PM100 leaf surface samples, in contrast to their representation in the PM10 communities.

While the PM10 communities of both zones and the PM100 from the construction zone showed similarities at the class level, differences were already apparent at the order level (and at lower taxonomic levels down to the species level). Thus, the PM10 communities retained significant similarities between them, whereas the PM100 from the leaves of the construction zone already differed from them and showed little similarity to the campus PM100 community (Figure S2).

##### Taxonomic Characterization of Fungal Communities

**Figure 5:**
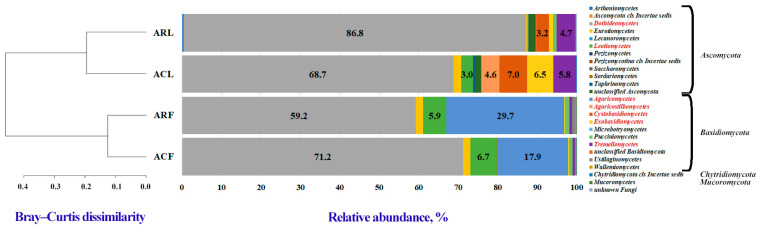
Distribution of the most abundant fungal classes identified in PM10 and PM100 collected in functional zones of Moscow. Unclassified classes and classes with relative abundance ≤ 0.1% were considered “unclassified” and “other”, respectively. Clustering analysis of the class-level communities based on the calculation of the pairwise Bray–Curtis dissimilarity matrix is also reported. ACF—PM10 of air from campus zone, ACL—PM100 of phylloplane from campus zone, ARF—PM10 of air from construction zone, ARL—PM100 of phylloplane from construction zone. Dominant classes in the sample bacterial communities are shown in red.

A total of four fungal phyla were identified from all air and leaf PM samples: *Ascomycota* (66.9–89.6%), *Basidiomycota* (10.1–32.8%), *Chytridiomycota* (0.00–0.06%), and *Mucoromycota* (0.00–0.11%). It should be noted that the phylum *Chytridiomycota* was not identified in PM10 samples of both zones, and the phylum *Mucoromycota* was not identified in PM100. *Dothideomycetes* (phylum *Ascomycota*) (59.2–86.8%), *Agaricomycetes* (phylum *Basidiomycota*) (0.1–29.7%), *Leotiomycetes* (phylum *Ascomycota*) (0.2–6.7%), and *Tremellomycetes* (phylum *Basidiomycota*) (0.6–5.8%) had the highest relative abundance among the 24 identified fungal classes (Figure 5, Table S2).

The fungal communities of the PM10 samples from the campus and the construction zone showed similarities in qualitative and relative abundance composition. The most abundant classes in these samples were *Dothideomycetes*, *Agaricomycetes*, and *Leotiomycetes*. The PM100 fungal communities of *Tilia* L. phylloplane of the construction zone and the campus differed at the class level in composition (presence of the *Arthoniomycetes* and *Saccharomycetes* classes in the PM100 road leaf sample, with relative abundances of 0.39% and 0.09%, respectively) and relative abundance of some classes. However, beta diversity analysis using the Bray–Curtis index showed that the fungal communities of PM100 leaves were similar to each other and different from those in PM10 air samples. *Dothideomycetes*, *Tremellomycetes*, and *Cystobasidiomycetes* had the highest relative abundance in the PM100 communities from the leaves. A distinctive feature of the community of PM100 from leaves of the campus zone was the high abundance of classes such as *Exobasidiomycetes* (phylum *Basidiomycota*) and *Agaricostilbomycetes* (phylum *Basidiomycota*) compared to communities from other samples.

A total of 59 fungal orders were identified in the four samples (Figure S3, Table S2). The highest relative abundance was observed for the orders *Dothideales* (phylum *Ascomycota*, class *Dothideomycetes*) (0.7–70.5%), *Cladosporiales* (phylum *Ascomycota*, class *Dothideomycetes*) (3.2–50.1%), *Pleosporales* (phylum *Ascomycota*, class *Dothideomycetes*) (2.1–14.9%), *Agaricales* (phylum *Basidiomycota*, class *Agaricomycetes*) (6.9–13.2%), *Polyporales* (phylum *Basidiomycota*, class *Agaricomycetes*) (0.1–11.6%), *Helotiales* (phylum *Ascomycota*, class *Leotiomycetes*) (0.1–6.7%), and *Filobasidiales* (phylum *Basidiomycota*, class *Tremellomycetes*) (0.2–5.2%).

The fungal communities of the leaf dust samples showed a similar pattern in the composition and relative abundance of some orders. A high relative abundance of the orders *Dothideales*, *Filobasidiales*, *Buckleyzymales*, and *Cladosporiales* was observed. A significant difference in the composition of the fungal communities of PM100 leaves was the higher content of the orders *Microstromatales* (phylum *Basidiomycota*, class *Exobasidiomycetes*) and *Agaricostilbomycetes*_ord_Incertae_sedis (phylum *Basidiomycota*, class *Agaricostilbomycetes*) in samples from the campus. The fungal communities of PM10 sampled from the construction zone and the campus had a similar composition at the order level (both in terms of order diversity and their relative abundance). Namely, the orders *Cladosporiales*, *Polyporales*, *Pleosporales*, *Helotiales*, and *Agaricales* had the highest relative abundance in both communities.

##### Analysis of Bacterial and Fungal Communities for Common ASVs

Venn diagrams (Figure S3) were constructed to show the similarity of the studied PM10 and PM100 communities (both bacterial and fungal) at the genus level. Among the four samples studied, the highest (192 genera) and the lowest (23 genera) number of unique taxa at the genus level were found in PM10 from the air (ACF) and PM100 from phylloplane (ACL) of the campus zone, respectively (Figure S4A). As shown in Figure S4A, the bacterial communities had 107 common ASVs at the genus level, representing 16.2%. Apparently, these elements represented the part of the bacterial community that circulates between the biotopes and functional zones of the studied zones of the RUDN sites due to air flows. It was also confirmed by the fact that the total number of ASVs for the PM10 communities without the common ones (16.2%) was 29.1%, and this value was 12.6 times higher compared to the data obtained for the PM100 communities. The bacterial genus *Pseudoxanthomonas* was detected only in the PM10 and PM100 samples collected from the construction zone. Three ASVs identified at the genus level (*Serratia*, *Phytohabitans*, and *Buttiauxella*) were detected only in PM100 regardless of sampling location.

Thirty-six common fungal ASVs at the genus level were found in the four samples studied (Figure S4B). The fungal community of PM10 from the construction zone had the highest number of unique genera. The number of common ASVs for the PM10 communities, excluding the common ones (11.3%), was 31.9%. For PM100, this value was 12.2%, which was 2.6 times lower than for PM10.

### 3.3. Functional Characterization of Bacterial and Fungal Communities in PM10 and PM100 Samples from the Construction Zone and Campus

**Figure 6:**
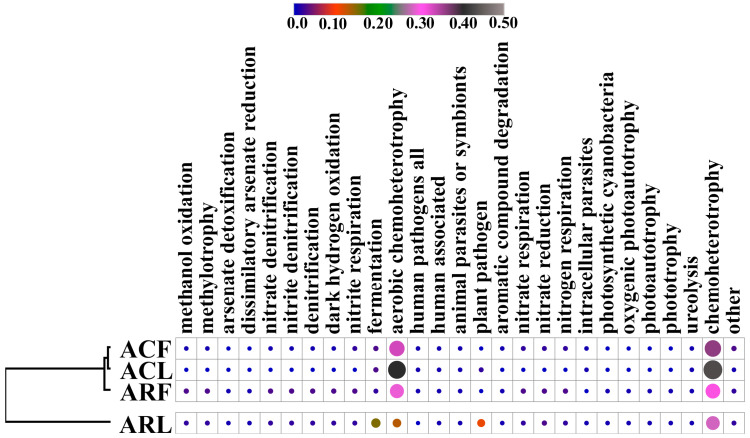
Heat maps of normalized data of relative abundance of ASVs (%) assigned to the functional groups bacterial identified species in microbiome of PM10 and PM100 collected in two functional zones of Moscow based on the FAPROTAX database. ACF—PM10 of air from campus zone, ACL—PM100 of phylloplane from campus zone, ARF—PM10 of air from construction zone, ARL—PM100 of phylloplane from construction zone.

In our work, we also analyzed the diversity of microbial feeding modes (predicted functional traits) contained in the microbial communities of air PM10 and leaf surface PM100. The FAPROTAX database allowed for the prediction of the presence of 55 functions in the studied bacterial communities with at least one record. It should be noted that there were only 27 predicted functional traits for which the relative abundance in the community was greater than 0.1%. Among all the groups detected, the relative abundances of Chemoheterotrophy and Aerobic Chemoheterotrophy were the highest in all the samples studied (Figure 6). The results of the hierarchical clustering showed that the bacterial communities of PM10 and PM100 sampled on the campus had common trends in grouping according to functional characteristics. Meanwhile, the PM10 and PM100 from the samples collected in the construction zone were significantly different.

**Figure 7:**
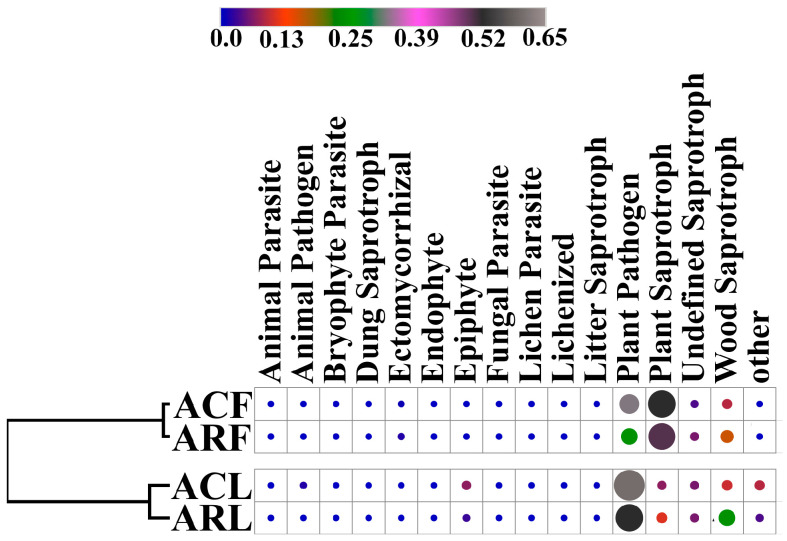
Heat maps of normalized data of relative abundance of ASVs (%) assigned to the functional groups fungal identified species in microbiome of PM10 and PM100 collected in functional zones of Moscow based on the FUNGuild database. ACF—PM10 from campus zone air, ACL—PM100 of leaf surface from campus zone, ARF—PM10 from construction zone air, ARL—PM100 of leaf surface from construction zone.

For the PM10 and PM100 fungal communities, 43 traits were predicted with at least one record (Figure 7). It should be noted that only 16 predicted functional traits had a relative abundance in the community greater than 0.1%. The Plant Saprotroph and Plant Pathogen groups had the highest relative abundances in all samples analyzed. The data obtained by calculating Pearson’s linear correlation coefficients and subsequent clustering of the samples showed that the functional characteristics of the fungal communities were grouped according to the sampling biotope (air and phylloplane).

### 3.4. Quantitative and Qualitative Assessment of PAHs and Metals in Selected PM10 and PM100

**Figure 8:**
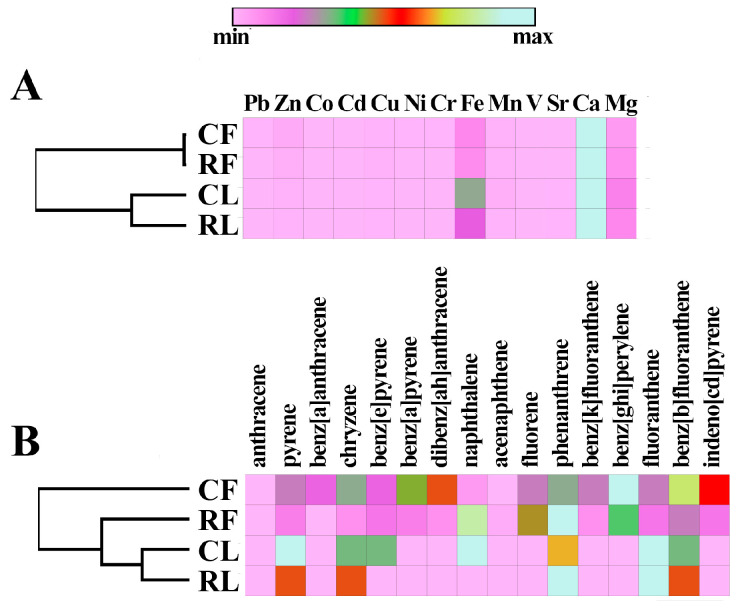
Heat maps of the metals (**A**) and PAHs (**B**) content in PM10 and PM100 of the different functional zones of Moscow. CF—PM10 from campus zone air, CL—PM100 of leaf surface from campus zone, RF—PM10 from construction zone air, RL—PM100 of leaf surface from construction zone.

In Table S3, we have summarised the quantitative and qualitative data on the content of metals and PAHs in the PM10 and PM100 samples. The contents of all investigated PAHs, Pb, Zn, Cd, and Cu, were predominant in PM10 samples compared to PM100. And the contents of Fe, Mn, Sr, Ca, and Mg were maximally represented in the composition of PM100. It should also be noted that the quantitative content of individual components in the samples differed between the sampling zones. For example, the concentrations of most of the PAHs were higher in the PM10 from the construction zone than in the campus PM10. The opposite trend was observed for the metals investigated. In addition, the concentrations of individual metals were also higher in the campus PM100 than in the construction zone PM100 (except for Ca). Heatmap and hierarchical clustering using Pearson’s coefficient showed that qualitative and quantitative metal contents were related to biotope type (data were clearly clustered by biotope type) (Figure 8). At the same time, the content of the investigated PAHs in the campus PM10 sample was significantly different from the other samples.

### 3.5. Correlation Analysis

**Figure 9:**
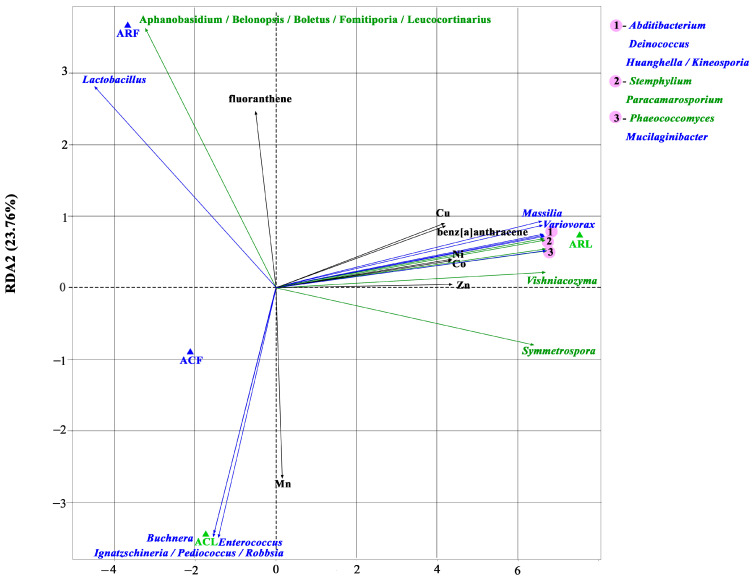
Triplot of RDA ordination of correlation showing correlations of PAHs and metals (black arrows), as well as bacterial (blue arrows) and fungal (green arrows) genera in PM10 and PM100 collected in functional zones of Moscow. Figures show only those bacterial and fungal genera, metals, and PAHs that were correlated where *p* < 0.05 after Bonferroni correction. ACF—PM10 of air from campus zone, ACL—PM100 of phylloplane from campus zone, ARF—PM10 of air from construction zone, ARL—PM100 of phylloplane from construction zone.

In the present study, metals and PAHs were some of the chemical compounds in PM10 and PM100 that could influence the formation of fungal and bacterial communities. Redundancy analysis (RDA) was used to assess the effect of metals and PAHs on microorganisms (Figure 9). RDA1 and RDA2 explained 69.36% and 23.76% of the variation in the samples, respectively.

Among the PAHs investigated, only benzanthracene and fluoranthene were found to correlate with bacterial and fungal genera. For metals, this was only found for Cu, Ni, Co, Zn, and Mn. Redundancy analysis showed that Cu, Ni, Co, Co, Zn, and benzanthracene were positively correlated with seven bacterial genera (*Massilia*, *Variovorax*, *Abditibacterium*, *Deinococcus*, *Huanghella*, *Kineosporia*, and *Mucliaginibacter*) and five fungal genera (*Vishnicozyma*, *Symmetrospora*, *Phaeococcomyces*, *Paracamarosporium*, and *Stemphylium*). Bacterial genera such as *Buchnera*, *Enterococcus*, *Ignatzschineria*, *Pediococcus*, and *Robbsia* were positively correlated with Mn and negatively correlated with fluoranthene. For the genera *Lactobacillus* (bacteria) and *Aphanobasidium*, *Belonopsis*, *Boletus*, *Fomitiporia*, and *Leucocortinarius* (fungi), an opposite trend was observed for these two types of pollutants.

**Figure 10:**
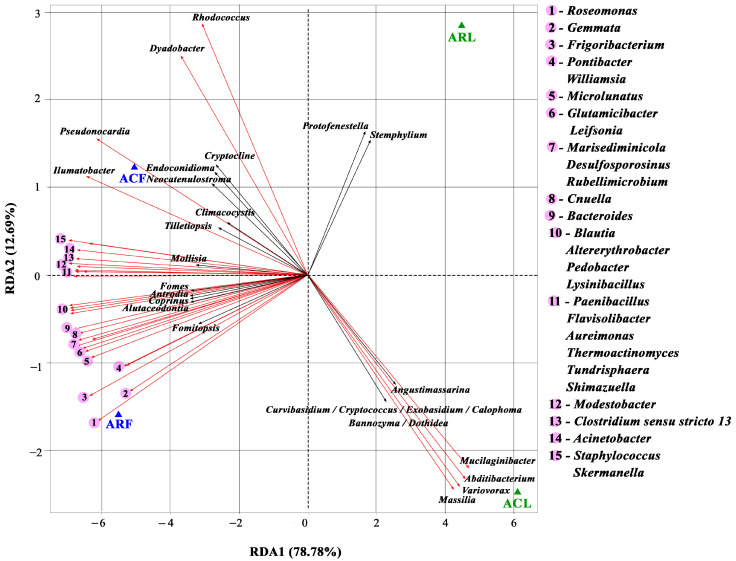
Triplot of RDA ordination showing the correlations of bacterial (red arrows) and fungal (black arrows) genera of PM10 collected in functional zones of Moscow. Figures show only those genera of fungi and bacteria that were correlated with each other with *p* < 0.05 after Bonferroni correction. ACF—PM10 of air from campus zone, ACL—PM100 of phylloplane from campus zone, ARF—PM10 of air from construction zone, ARL—PM100 of phylloplane from construction zone.

RDA was also performed to assess the interaction between members of the bacterial and fungal communities (Figure 10). RDA1 and RDA2 explained 78.78% and 12.69% of the variation in the samples, respectively. Of the 484 bacterial genera and 287 fungal genera identified in the microbial communities, only 35 and 20 bacterial and fungal genera, respectively, were found to be correlated with each other. Most genera showed both positive and negative correlations. The exceptions were *Protofenestella* and *Stemphylum*. These two genera of fungi only showed a negative correlation with other members of the microbial community (Figure 10).

## 4. Discussion

In the present study, air PM10 and PM100 from the surface of *Tilia* L. leaves were compared in the campus and construction zone of Moscow near the RUDN campus in autumn. According to previous studies [40,41], the functional zoning of the city (especially the difference between conditionally clean and traffic zones) did not contribute significantly to changes in the microbial communities of paved surfaces and *Betula* L. phylloplane, in contrast to the effects of pollutants. It was interesting to compare the effects of pollution on the air microbiome of zones with different levels of anthropogenic pollution and dust particles deposited on the leaves of the woody plant *Tilia* L. in the air sampling zone. *Tilia* L. was chosen as a woody plant that can be used for air characterization due to its biological properties. Under conditions of anthropogenic pollution, a tree is capable of absorbing heavy metals through its leaves, branches, and fruits acting as their concentrator, thus purifying the atmospheric air from toxic elements [42]. The leaves of *Tilia L.* are particularly good at absorbing zinc and lead, the accumulation coefficient of which is directly proportional to the level of air pollution. This indicator allows researchers to determine the difference in the level of air pollution by heavy metals when comparing different parts of the city. In our study, we obtained similar results not only for zinc and lead, but also for such metals as cobalt, copper, and nickel. The high levels of calcium, magnesium, and iron in PM100 were not so much related to the accumulative effect of dust particles on the surface of leaves but rather to the role of these elements in plant life activity, including photosynthesis [43,44,45]. In addition, leaves can be used to estimate the amount of dust in the air [46]. For example, in a study conducted in 2021 at several sites in Haidian District (China) with different traffic loads, the amount of PM on leaves was found to be several times higher under polluted conditions compared to the control [47]. The structure of the leaf surface is a critical factor in a plant’s ability to retain PM. Lint, stomata, and grooves on the leaf surface can increase its roughness, resulting in more efficient PM retention. Our study showed that the air near the construction zone contained higher levels of PM, including spherical particles. It is well known that anthropogenic particles emitted from combustion processes have a spherical and round shape with a smooth surface [48]. We also detected soot (black carbon) particles on the adaxial side of the leaves of *Tilia* L. growing near the construction zone. Soot is the primary PM emitted into the air as a result of industrial processes, power plants, traffic, etc. [33,49]. In recent years, researchers have focused on analyzing the ability of different trees to accumulate black carbon in order to recommend tree species for urban landscaping in the future [50,51,52]. Obviously, such a pollutant affects the microbial communities of the phylloplane. This was probably the reason why microbial cells associated with PM100 were only found on the surface of *Tilia* L. leaves in the campus area, where the air was cleaner.

According to our data, most of the analyzed PAHs were found in the air of the construction zone, but they were not detected in the PM100 of this zone. This may be due to the fact that the surface of leaves provides a more favorable environment for the growth and development of microorganisms [20]. It should also be noted that a characteristic feature of the plant cell is the formation of phenolic compounds [53]. Thus, the phylloplane microbial community was not only already adapted to the presence of benzene-containing compounds but also had the ability to use additional nutrients to break down aromatic compounds. Therefore, PAHs deposited on the leaf surface could be metabolized by microbial degraders. It appears that microbial communities are more dynamic in the composition of atmospheric particulate matter. This may result in a lack of time for the formation of a stable microbial community capable of rapidly degrading PAHs. Another example is phylloplane microbial communities, where rapid PAH degradation can be provided by several groups of epiphytic microorganisms, with some members of the community degrading complex PAHs to simpler forms, while other members (e.g., commensals) rapidly consume these simple PAHs as an energy source. It is possible that the levels of PAHs in the cells and tissues of leaves (i.e., directly inside the plant) collected in the construction zone are higher than in leaves from the campus due to the uptake of gaseous PAHs. However, as our study focused on dust particles, we did not investigate this level of PAHs in the leaves themselves.

Air is an unfavorable environment for microorganisms (low nutrients, UV radiation, low humidity, etc.). However, the alpha diversity indices of both bacterial and fungal communities in air aerosols were higher than those of PM100 communities. This may be due to the large and temporal heterogeneity of PM10 communities caused by constantly moving air flows and dust fractions from paved surfaces or the top soil layer entering the air. The latter two are known to be characterized by higher biodiversity than phyllosphere communities [54]. Although the communities of the two biotopes (both bacterial and fungal) differed from each other based on the Bray–Curtis dissimilarity matrix, the same elements were found at the genus level (ASVs). Apparently, the microorganisms belonging to these ASVs circulated between the biotopes and the study zones precisely because of the PM in the air. However, in general, their contribution to the microbiome of PM100 was insignificant to cause changes in the phylloplane microbial communities and to show greater similarity between the communities of the two biotopes. It should be noted that plants can secrete phytoncides, which play an important role in plant immunity and in the relationships between organisms within an ecosystem. These compounds may contribute to the formation of a certain composition of the microbial community (predominance of some taxa and inhibition of others). On the other hand, the phylloplane communities themselves may already be established (again due to the compounds released by the plant and the influence of pollutants on the ecosystem as a whole). As a result, when microorganisms from outside enter their environment, they may gradually outcompete them. Our data showed that the bacterial communities of PM100 from the leaves of the construction zone were significantly influenced by anthropogenic factors, in contrast to the fungal communities. Apparently, there was an additional factor in the construction zone that was critical for the phylloplane bacterial community but did not (or only slightly affected) affect the fungal community. The phylloplane bacteria could also become more sensitive to environmental pollutants indirectly through changes in soil chemistry [55]. The data obtained in our current study on the predicted metabolic activity pathways can also be related to this. The soil under the trees in the study area is under constant stress from construction machinery and the transport of materials. Another factor is the cleaning of the pavements and carriageways of Moscow’s main roads. This appears to have caused the phylloplane bacterial community in the construction zone to differ significantly in metabolic functions from the other three.

Air and phylloplane have different habitat conditions and food sources, which is particularly important for micromycetes. Aridity, increased UV radiation, and the lack of nutrients characteristic of the air mean that xerophilous fungi, which include members of the phylum *Mucoromycota*, can be found in this biotope [56]. At the same time, members of the phylum *Chytridiomycota* were found only in the phylloplane. This is not surprising. Plant pathogens have been identified among them. In addition, *Chytridiomycota* are able to grow in environments with high levels of moisture [57]. Such conditions are certainly characteristic of phylloplane due to the presence of additional food sources, namely plant excretions and the presence of moisture.

Microorganisms within the community interact and influence each other in one way or another. Fungi play an important role in the microbial community, and interactions between individual fungi and bacteria have been well documented [58]. The relationship between bacteria and fungi can be symbiotic (bacteria can have a beneficial effect on fungi), parasitic (bacteria can grow by destroying live fungal hyphae), and competitive (bacteria can compete with fungi for nutrient substrate, and their competitiveness is enhanced by the release of antibiotics and toxins). For example, in the case of manganese-oxidising bacteria of the genus *Metallogenium*, bacteria use hydrogen peroxide produced by fungi to oxidise manganese, and members of the genera *Bacillus*, *Paenibacillus* and *Streptomyces* can be parasitic on fungi [59]. In addition, fungi release volatile organic compounds that are perceived by bacteria as signals to become more active and prepare for the action of antibiotics (they begin to actively synthesise proteins to move in space, thicken their cell walls, and divide more actively) [60]. Evaluation of the interactions between these two domains in the microbial communities studied in our work showed that the ratio of bacterial community members dependent on fungal community members was 5–10 times higher. In addition, 12–13% of the bacterial community of phylloplane correlated with a number of the metals and PAHs studied, in contrast to the fungal community (0.4–2.6%). Thus, the bacterial communities of the studied biotopes appeared to be more sensitive to the selected pollutants. A similar assumption was made in the work of Li et al. [61]. Furthermore, Fan et al. [62] found that smog levels had a more pronounced effect on bacterial pathogens and ammonia-oxidising microorganisms than on fungi.

Different groups of microorganisms react differently to heavy metals, depending on the type and concentration of heavy metals in the environment [63]. On the one hand, some are essential in small quantities [43,44,45]. On the other hand, high concentrations of metals can have different effects on bacteria and fungi. In bacteria, high concentrations of heavy metals can inhibit cell division, reduce enzyme activity and translation, denature proteins, and damage DNA and cell membranes [64]. This can lead to a decrease in the abundance of bacteria in the biotope. At the same time, heavy metal pollution can both suppress the community of microscopic fungi and stimulate their development. For example, strontium can increase toxin formation and stimulate the growth of fungi of the genus *Fusarium* [65]. In general, micromycetes are more resistant to heavy metals than bacteria due to the presence of specialised proteins, metallothioneins, which bind large amounts of metals [66,67].

Like heavy metals, bacteria are more sensitive to polycyclic aromatic hydrocarbons. This may be due, for example, to the presence of a special sensory system that provides a rapid response to the presence of PAHs by directly controlling sequential responses, including chemotactic perception and movement [68]. In contrast, micromycetes often exhibit resistance to hydrocarbons due to their abundant spore formation and specific enzyme systems [69].

In general, anthropogenic influences, including the presence of metals and polycyclic aromatic hydrocarbons, affect the structure of microbial communities, stimulating the survival and development of species with high levels of resistance to these pollutants.

## 5. Conclusions

This study evaluated the influence of anthropogenic factors on the diversity of microbial communities of PM10 air and PM100 of *Tilia* L. phylloplane in the urban ecosystem of Moscow. Similarities and differences between air and phylloplane microbial community structures in different functional zones of the city were investigated. Air and phylloplane bacterial communities were found to be more sensitive to environmental pollution than fungal communities. This was particularly evident in the case of phylloplane. If we consider phylloplane bacterial communities as an indicator of air quality, it is necessary to compare the phylloplane bacterial communities of different plants. In this case, it is also necessary to exclude the influence of the host plant on the microbiome formed. In addition, the phylloplane of the selected plant should have good deposition properties with respect to pollutants. Therefore, further research should be directed towards a comparative evaluation of the deposition properties and microbial communities of the phylloplane of different woody species in Moscow.

## Data Availability

Dataset available on the NCBI SRA Portal; the accession numbers are PRJNA1142405 (bacterial and fungal communities). The name of the project is “RUDN _2023Dust”.

## Supplementary Materials

The following supporting information can be downloaded at: https://www.mdpi.com/article/10.3390/biology13120969/s1, Figure S1. Overview of PM10 and PM100 sampling sites: 1—woody plant growing on the campus, 2—air PM10 sampled in the campus zone using an air sampler, 3—woody plant growing near the construction zone, 4—air PM10 sampled in the construction zone; Figure S2. Distribution of the most abundant bacterial orders identified in PM10 and PM100 collected in two zones of RUDN. Unclassified orders and orders with relative abundance ≤ 0.1% were considered “unclassified” and “other”, respectively. Clustering analysis of the class-level communities based on the calculation of the pairwise Bray–Curtis dissimilarity matrix is also reported; Figure S3. Distribution of the most abundant fungal orders identified in PM10 and PM100 collected in two zones of RUDN. Unclassified orders and orders with relative abundance ≤ 0.1% were considered “unclassified” and “other”, respectively. Clustering analysis of the class-level communities based on the calculation of the pairwise Bray–Curtis dissimilarity matrix is also reported; Figure S4. Venn diagrams illustrating the number of unique and shared bacterial and fungal ASVs among PM10 and PM100 from Moscow detected in two zones (campus and construction zone) in autumn. A—bacterial genera; B—fungal genera; Table S1. Relative abundance of bacterial classes and orders; Table S2. Relative abundance of fungal classes and orders; Table S3. Quantitative and qualitative content of metals and PAHs in PM10 and PM100.
