# Supplementary material for: Impact of Anthropogenic Factors on the Diversity of Microbial Communities of PM10 Air and PM100 of Tilia L. Phylloplane in an Urban Ecosystem
Source: Biology (Basel). 2024 Nov 24;13(12):969. doi: 10.3390/biology13120969 (PMC11673261; doi:10.3390/biology13120969)
Supplement: Supplementary file 1 [file biology-13-00969-s001.zip › biology-3309103-supplementary/Supplementary materials_biology-3309103_new/Supplementary materials_new.pdf]

Supplementary material.

Olesya I. Sazonova[1,*], Anastasia A. Ivanova[1], Anna A. Vetrova[1], Anton N. Zvonarev[1], Rostislav A. Streletskii[2], Viacheslav I. Vasenev[3,4], Vladimir A. Myazin[3,5], Ksenia I. Makhinya[3], Ekaterina V. Kozlova[3], and Maria V. Korneykova[3,5]

[1] Federal Research Center "Pushchino Scientific Center for Biological Research of the Russian Academy of Sciences", 142290 Pushchino, Russia; mrs.ivanova.a.a@gmail.com (A.A.I.), (A.N.Z.)
[2] Faculty of Soil Science, Laboratory of Ecological Soil Science, Lomonosov Moscow State University, 119991 Moscow, Russia; streletskiyrostislav@mail.ru (R.A.S.)
[3] Peoples' Friendship University of Russia (RUDN), 117198 Moscow, Russia; myazinv@mail.ru (V.A.M), makhinya-ki@rudn.ru (K.I.M), kozlova-ev@rudn.ru (E.V.K)
[4] Soil Geography and Landscape group, Wageningen University, 6707, Wageningen, Netherlands; slava.vasenev@wur.nl (V.I.V.)
[5] Institute of North Industrial Ecology Problems Subdivision of the Federal Research Center "Kola Science Centre of Russian Academy of Science", 184209 Apatity, Russia
*Correspondence: sazonova_oi@rambler.ru (O.I.S.); korneykova.maria@mail.ru (M.V.K)

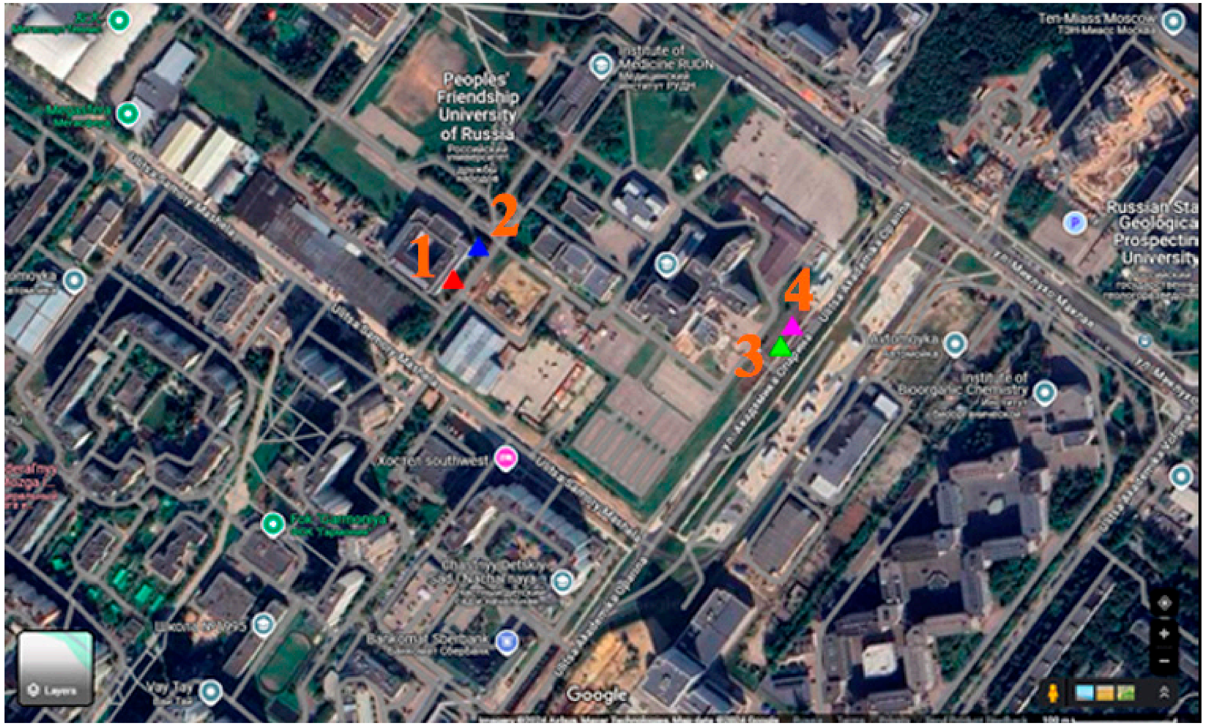

Figure 1S. Overview of PM10 and PM100 sampling sites. 1 - woody plant growing on the campus, 2 - air PM10 sampled in the campus zone using an air sampler, 3 - woody plant growing near the construction zone, 4 - air PM10 sampled in the construction zone.

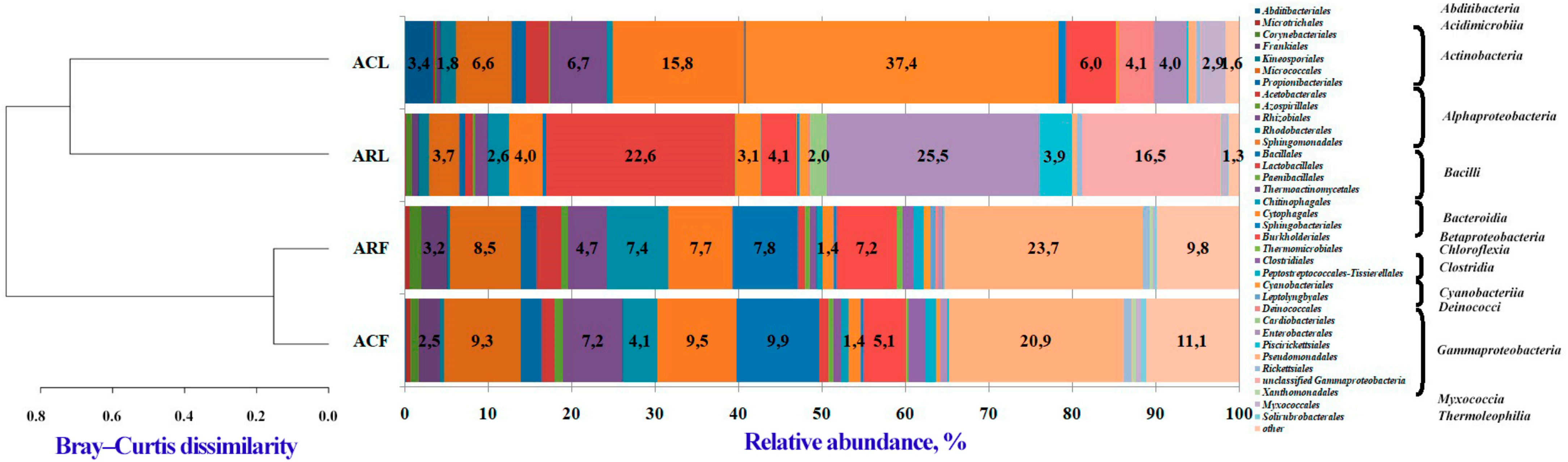

Figure 2S. Distribution of the most abundant bacterial orders identified in PM10 and PM100 collected in two zones of RUDN. Unclassified orders and orders with relative abundance ≤0.1% were considered as "unclassified" and "other", respectively. Clustering analysis of the class-level communities based on the calculation of the pairwise Bray-Curtis dissimilarity matrix is also reported.

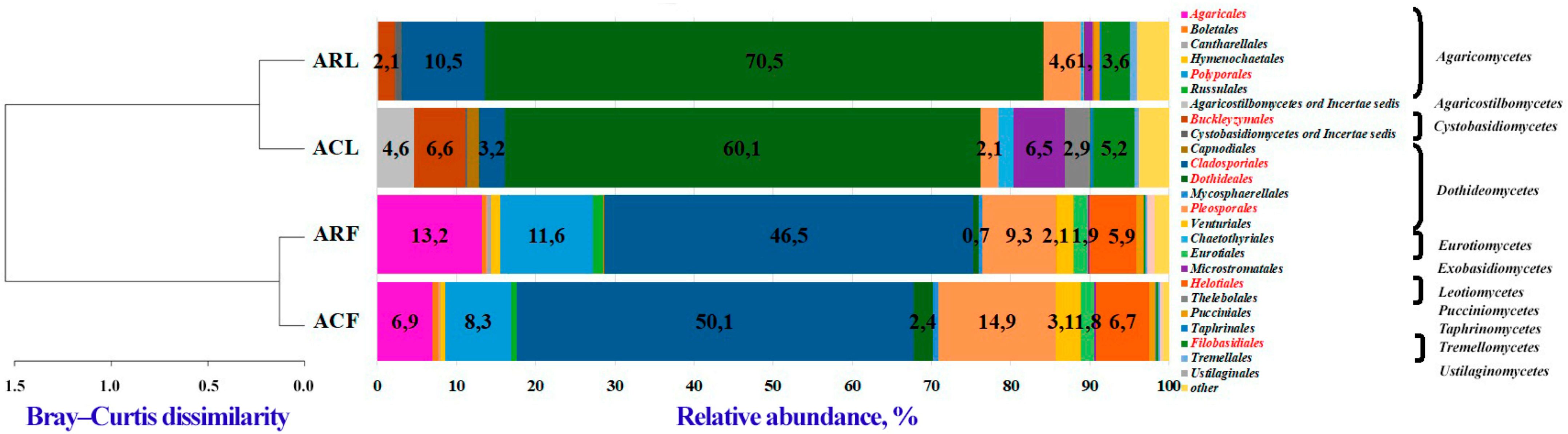

Figure 3S. Distribution of the most abundant fungal orders identified in PM10 and PM100 collected in two zones of RUDN. Unclassified orders and orders with relative abundance ≤0.1% were considered as "unclassified" and "other", respectively. Clustering analysis of the class-level communities based on the calculation of the pairwise Bray-Curtis dissimilarity matrix is also reported.

Table 1S. Relative abundance of bacterial classes and orders

Class	Order	ACF	ARF	ACL	ARL
Abditibacteria	*Abditibacteriales*	0.1	0.1	3.4	0.1
Acidimicrobiia	*Microtrichales*	0.6	0.4	0.1	0.1
Actinobacteria	*Corynebacteriales*	1.0	1.4	0.2	0.7
	Frankiales	2.5	3.2	0.5	0.8
	Kineosporiales	0.4	0.2	1.8	1.2
	Micrococcales	9.3	8.5	6.6	3.7
	Propionibacteriales	2.4	1.9	1.8	0.7
Alphaproteobacteria	*Acetobacterales*	1.6	3.0	2.7	0.9
	Azospirillales	1.0	0.7	0.2	0.2
	Rhizobiales	7.2	4.7	6.7	1.5
	Rhodobacterales	4.1	7.4	0.7	2.6
	Sphingomonadales	9.5	7.7	15.8	4.0
Bacilli	*Bacillales*	9.9	7.8	0.1	0.5
	Lactobacillales	1.0	0.8	0.2	22.6
	Paenibacillales	0.6	0.7	0.0	0.0
	Thermoactinomycetales	1.0	0.7	0.0	0.0
Bacteroidia	*Chitinophagales*	0.9	0.7	0.0	0.0
	Cytophagales	1.4	1.4	37.4	3.1
	Sphingobacteriales	0.4	0.3	0.9	0.1
Betaproteobacteria	*Burkholderiales*	5.1	7.2	6.0	4.1
Chloroflexia	*Thermomicrobiales*	0.3	0.7	0.0	0.1
Clostridia	*Clostridiales*	2.0	1.4	0.0	0.1
	Peptostreptococcales-Tissierellales	1.3	1.2	0.0	0.2
Cyanobacteriia	*Cyanobacteriales*	0.4	0.8	0.3	1.0
	Leptolyngbyales	0.0	0.6	0.0	0.0
Deinococci	*Deinococcales*	0.1	0.3	4.1	0.3
Gammaproteobacteria	*Cardiobacteriales*	0.0	0.0	0.0	2.0
	Enterobacterales	0.8	0.6	4.0	25.5
	Piscirickettsiales	0.2	0.2	0.2	3.9
	Pseudomonadales	20.9	23.7	1.0	0.6
	Rickettsiales	0.9	0.8	0.5	0.6
	unclassified Gammaproteobacteria	0.1	0.0	0.1	16.5

	Xanthomonadales	0.5	0.4	0.0	0.2
Myxococcia	*Myxococcales*	0.6	0.2	2.9	0.7
Thermoleophilia	*Solirubrobacterales*	0.6	0.3	0.0	0.1
other		11.1	9.8		1.3

Table 2S. Relative abundance of fungal classes and orders

Class	Order	ACF	ARF	ACL	ARL
Agaricomycetes	*Agaricales*	6.9	13.2	0.0	0.0
	Boletales	0.9	0.6	0.0	0.0
	Cantharellales	0.2	0.5	0.0	0.0
	Hymenochaetales	0.7	1.2	0.0	0.0
	Polyporales	8.3	11.6	0.0	0.1
	Russulales	0.6	1.5	0.0	0.0
Agaricostilbomycetes	*Agaricostilbomycetes ord Incertae sedis*	0.0	0.0	4.6	0.0
Cystobasidiomycetes	*Buckleyzymales*	0.0	0.1	6.6	2.1
	Cystobasidiomycetes ord Incertae sedis	0.0	0.0	0.2	0.9
Dothideomycetes	*Capnodiales*	0.0	0.0	1.5	0.0
	Cladosporiales	50.1	46.5	3.2	10.5
	Dothideales	2.4	0.7	60.1	70.5
	Mycosphaerellales	0.7	0.5	0.0	0.0
	Pleosporales	14.9	9.3	2.1	4.6
	Venturiales	3.1	2.1	0.0	0.0
Eurotiomycetes	*Chaetothyriales*	0.0	0.0	2.0	0.3
	Eurotiales	1.8	1.9	0.0	0.1
Exobasidiomycetes	*Microstromatales*	0.2	0.2	6.5	1.1
Leotiomycetes	*Helotiales*	6.7	5.9	0.1	0.1
	Thelebolales	0.0	0.0	2.9	0.1
Pucciniomycetes	*Pucciniales*	0.8	0.9	0.1	0.7
Taphrinomycetes	*Taphrinales*	0.1	0.1	0.5	0.3
Tremellomycetes	*Filobasidiales*	0.3	0.2	5.2	3.6
	Tremellales	0.2	0.2	0.5	0.9
Ustilaginomycetes	*Ustilaginales*	0.4	0.9	0.0	0.0
other		0.8	1.8	3.8	4.0

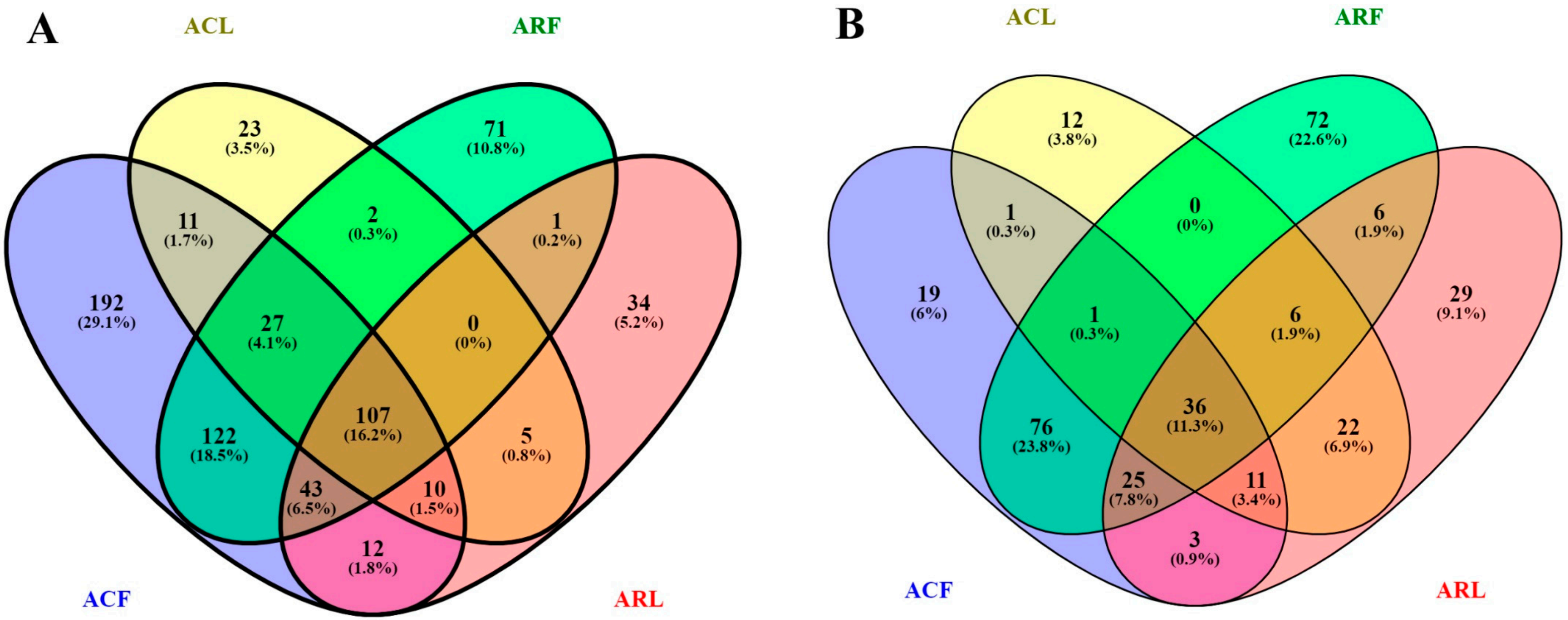

Figure 4S. Venn diagrams illustrating the number of unique and shared bacterial and fungal ASVs among PM10 and PM100 from Moscow detected in two zones (campus and construction zone) in autumn. A – bacterial genera; B – fungal genera

Table 3S. Quantitative and qualitative content of metals and PAHs in dust particles

Pollutants	mg/g PM10		mg/g PM100	
	**ARF**	**ACF**	**ARL**	**ACL**
anthracene	0.001	0.000	0.000	0.000
pyrene	0.007	0.004	0.001	0.003
benz[a]anthracene	0.001	0.003	0.000	0.000
chryzene	0.005	0.005	0.001	0.001
benz[e]pyrene	0.008	0.003	0.000	0.001
benz[a]pyrene	0.007	0.007	0.000	0.000
dibenz[ah]anthracene	0.005	0.008	0.000	0.000
naphthalene	0.040	0.001	0.000	0.003
acenaphthene	0.002	0.000	0.000	0.000
fluorene	0.023	0.004	0.000	0.000
phenanthrene	0.049	0.005	0.002	0.002
benz[k]fluoranthene	0.005	0.004	0.000	0.000
benz[ghi]perylene	0.018	0.016	0.000	0.000
fluoranthene	0.008	0.004	0.002	0.003
benz[b]fluoranthene	0.013	0.012	0.001	0.001
indeno[cd]pyrene	0.008	0.009	0.000	0.000
Pb	0.1050	0.3938	0.0076	0.1036
Zn	1.8675	5.6063	1.7434	2.6005
Co	0.0000	0.0281	0.0000	0.0011
Cd	0.0293	0.0534	0.0000	0.0000
Cu	0.7500	1.4063	0.6097	0.6146
Ni	0.0000	0.3750	0.0000	0.0114
Cr	0.0081	0.3227	0.1673	0.1735
Fe	9.0000	23.2500	62.0007	84.8677
Mn	0.9750	2.1563	2.0197	4.3930
V	0.0912	0.0000	0.0000	0.0000
Sr	0.0225	0.0750	0.6650	0.6874
Ca	95.2500	220.3125	303.7158	275.5328
Mg	7.5000	14.0625	31.2481	32.4357
