# Supplementary figures and images for: Impact of Anthropogenic Factors on the Diversity of Microbial Communities of PM10 Air and PM100 of Tilia L. Phylloplane in an Urban Ecosystem
Source: Biology (Basel). 2024 Nov 24;13(12):969. doi: 10.3390/biology13120969 (PMC11673261; doi:10.3390/biology13120969)

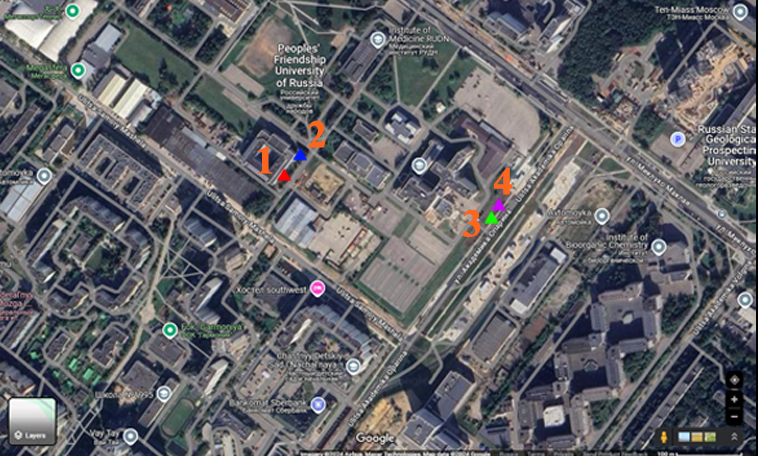

Supplement: Supplementary file 1 [file biology-13-00969-s001.zip › biology-3309103-supplementary/Supplementary materials_biology-3309103_new/Figure 1S.jpg]

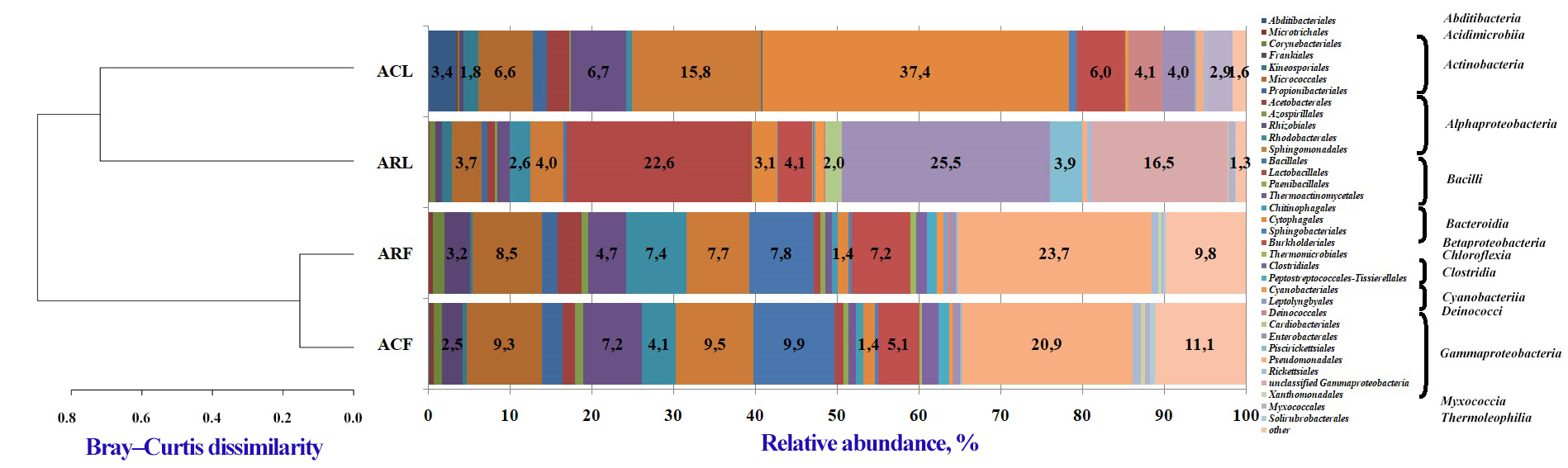

Supplement: Supplementary file 1 [file biology-13-00969-s001.zip › biology-3309103-supplementary/Supplementary materials_biology-3309103_new/Figure 2S.jpg]

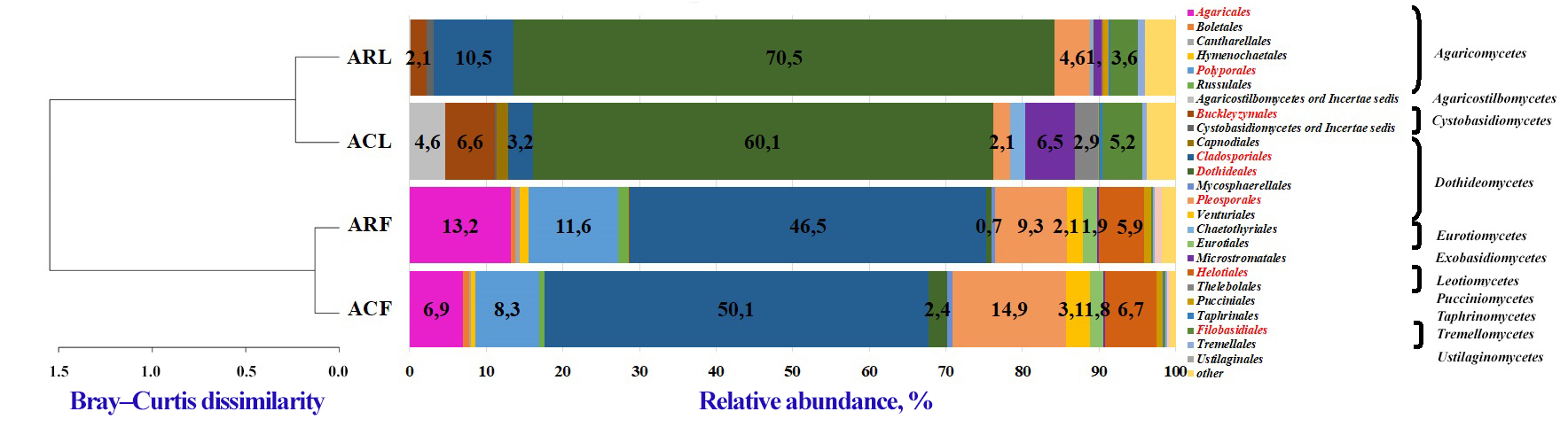

Supplement: Supplementary file 1 [file biology-13-00969-s001.zip › biology-3309103-supplementary/Supplementary materials_biology-3309103_new/Figure 3S.jpg]

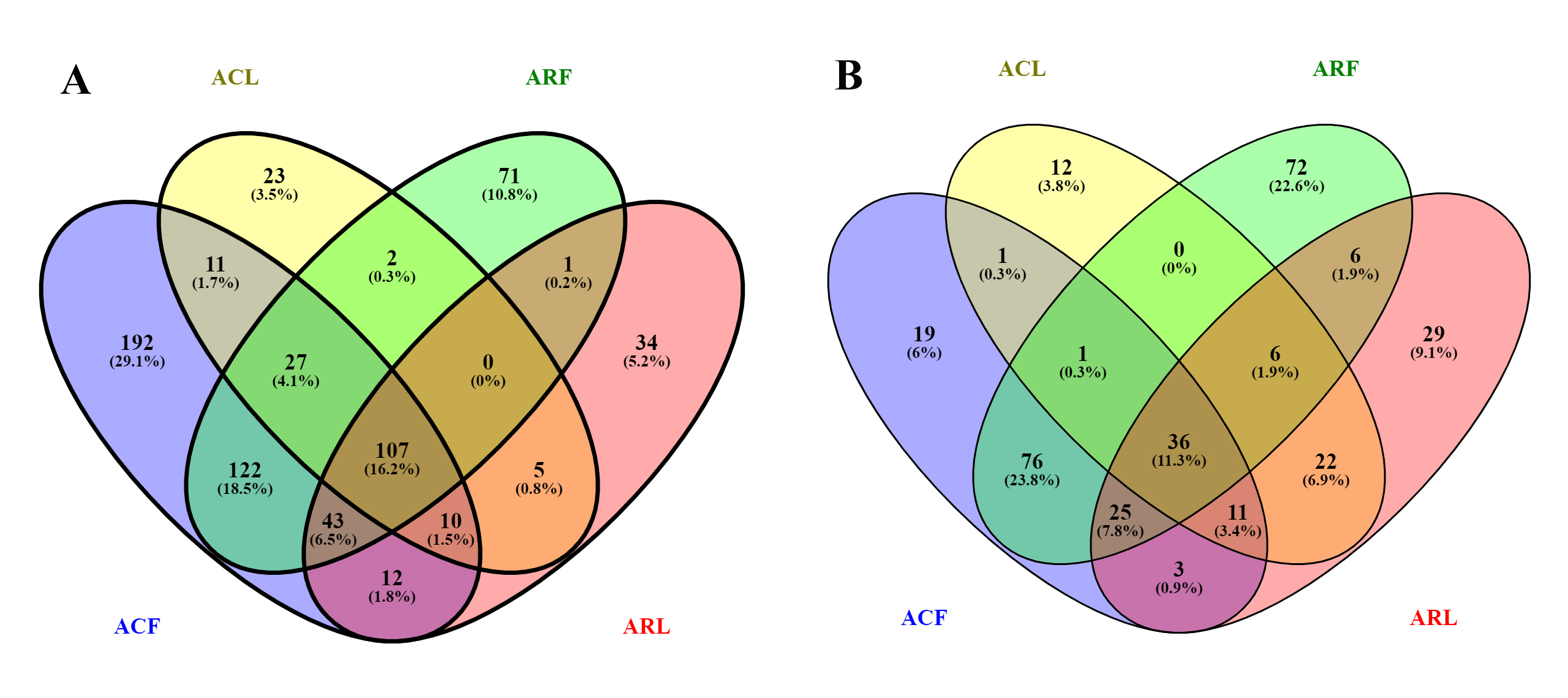

Supplement: Supplementary file 1 [file biology-13-00969-s001.zip › biology-3309103-supplementary/Supplementary materials_biology-3309103_new/Figure 4S.jpg]
